# Metronomics as Maintenance Treatment in Oncology: Time for Chemo-Switch

**DOI:** 10.3389/fonc.2014.00076

**Published:** 2014-04-10

**Authors:** Prabhat Singh Malik, Vinod Raina, Nicolas André

**Affiliations:** ^1^Department of Medical Oncology, Dr. BRAIRCH, All India Institute of Medical Sciences, New Delhi, India; ^2^Metronomics Global Health Initiative, Marseille, France; ^3^Fortis Memorial Research Institute, Gurgaon, India; ^4^Service d’Hématologie et Oncologie Pédiatrique, AP-HM, Marseille, France; ^5^INSERM UMR 911, Centre de Recherche en Oncologie Biologique et Oncopharmacologie, Aix-Marseille University, Marseille, France

**Keywords:** metronomic chemotherapy, maintenance, chemo-switch, global perspective, low-dose chemotherapy

The “cell kill” paradigm associated with maximum-tolerated dose (MTD) chemotherapy has shown remarkable success in many hematological malignancies, but unfortunately has failed to demonstrate sustained responses in majority of common advanced solid tumors. This failure can be attributable to heterogeneity of cancer cells within the tumor in terms of the rate of cell proliferation, genetic makeup, and micro-environmental selection giving rise to development of treatment resistance. In this context, Gatenby’s hypothesis of controlling tumor growth rather than eradicating it and treating it like a chronic disease may be more meaningful (1). The use of metronomic regimens alone or in combination with other new targeted therapies after MTD chemotherapy as maintenance treatment for longer period may pave the way for long-term cost-effective treatment.

## Metronomic Chemotherapy

Metronomic chemotherapy (MC), a term initially coined by Hanahan, is defined as administration of chemotherapeutic agents at relatively low, minimally toxic doses, and with no prolonged drug-free breaks (2). This concept originated by pioneer work done by Klement et al. and Browder et al. showing that mice bearing subcutaneous tumors could respond to frequent repeated low doses of chemotherapy, even when they displayed acquired drug resistance to the same agents given in a conventional way (3, 4).

Several mechanisms of action have since been proposed for MC, the major being the inhibition of angiogenesis by selective inhibition of proliferation and/or induction of apoptosis of activated endothelial cells, selective inhibition of endothelial cell migration, increase in the expression level of the endogenous angiogenesis inhibitor thrombospondin-1, and sustained decrease in levels and viability of bone marrow-derived endothelial progenitor cells (5). In addition, MC has immuno-modulatory effect through Treg depletion, NK cell and T cell activation, and promoting dendritic cell activation (5, 6). Overall, these properties may lead to re-induction of tumor dormancy. Therefore, MC can be regarded as an intrinsically multi-targeted therapy (5, 7).

The use of MC in the clinic practice has been mainly limited to palliative purposes in relapse/refractory diseases, with good response rates and a favorable toxicity profile. In a recent systemic review of 80 small single arm and phase II studies of low-dose MC in various tumor types including 3700 patients, Lien et al. showed mean response rates of 26%, median progression-free survival (PFS) of 4.6 months, and mean disease control rates of 56%. Grade 3/4 adverse events were found to be rare (anemia 8%, fatigue 13%) (8). Building on these encouraging results, nine phase III trials are currently registered at ClinicalTrials.gov in various clinical settings including adjuvant and maintenance.

## Maintenance Treatment: Scope in Various Solid Tumors

The U.S. National Cancer Institute’s (NCI’s) medical dictionary defines maintenance therapy as “*type of treatment that is given to prevent progression after it has been controlled successfully with the initial therapy*.” It includes treatment with drugs, vaccines, or antibodies with anticancer properties and may be given for extended period of time. Aims of treatment in many advanced cancers are to prolong survival and improve patient’s quality of life. An effective maintenance therapy should seek to achieve both of these goals with a good patient tolerance, lack of cumulative toxicities, and cost–effectiveness. In this context, two treatment paradigms have emerged; continuation maintenance where one component of initial therapy is continued after the induction treatment and switch maintenance in which a new and potentially cross-resistant agent is introduced (9).

Maintenance therapy has been classically used for hematological malignancies like acute lymphoblastic leukemia (ALL) (10, 11) and low-grade non-Hodgkin’s lymphoma (12, 13). Recently, there has been a growing interest to explore the role of maintenance in various advanced solid tumors. Advanced non-small cell lung cancer (NSCLC), metastatic colorectal cancer (CRC), metastatic breast cancer, and ovarian cancer are few examples where maintenance treatments with either chemotherapy or targeted therapies have shown promising results. In NSCLC, most of the strategies of either continuation or switch maintenance demonstrated a significant improvement in PFS except for pemetrexed and erlotinib which also showed an overall survival (OS) improvement (9). A recent meta-analysis incorporating data of 3736 patients from eight randomized controlled trials on maintenance treatment in advanced NSCLC altogether suggest, that OS and PFS are clearly in favor of maintenance therapy for both, switch and continuation strategy (14). Similarly for metastatic breast cancer, a meta-analysis including 2269 patients from 11 randomized trials showed a clear benefit of longer duration of chemotherapy in terms of PFS and OS (15). Several studies in metastatic CRC have demonstrated improvement in survival by continuing part of the induction chemotherapy and/or targeted agents beyond six cycles (16–18). The role of maintenance chemotherapy in ovarian carcinoma has been more controversial, different large randomized trials showing variable results (19, 20). Recent meta-analysis of 1644 patients from eight trials showed no benefit of maintenance chemotherapy in ovarian carcinoma (21). However, anti-angiogenic agents like bevacizumab have shown promising activity when combined with chemotherapy and continued as maintenance (22). In a recent phase II study, olaparib, an oral poly[adenosine diphosphate (ADP)–ribose] polymerase inhibitor has also shown benefit in terms of PFS when used as a maintenance therapy in platinum-sensitive relapse ovarian cancers (23). Results of an international Intergroup trial (AGO-OVAR16) presented at annual conference of American Society of Clinical Oncology (ASCO) in 2013 demonstrated a PFS benefit of pazopanib maintenance in advanced epithelial ovarian cancer (24).

## Economic and Quality of Life Considerations with Maintenance Treatment

Cost–effectiveness and quality of life are two other major issues associated with prolonged use of maintenance therapy. The economic impact of maintenance for advanced cancers has been studied in numerous cost–effectiveness analyses. For example, the incremental cost–effectiveness ratio (ICER) per life-year gained with maintenance pemetrexed in advanced NSCLC was estimated to be $205,597 (25). Similarly, in ovarian cancer, economic evaluation of maintenance paclitaxel and maintenance bevacizumab demonstrated that compared with carboplatin–paclitaxel for six cycles, maintenance paclitaxel had an ICER of $13,402 per quality-adjusted life-year while addition of bevacizumab to carboplatin–paclitaxel and then as maintenance monotherapy had an ICER of $326,530 per quality-adjusted life-year (26). In a systematic review, addition of bevacizumab to chemotherapy in metastatic CRC was also found not to be cost-effective (27).

Tolerance and compliance of chronic treatment are major concerns but second line therapy on progression can also lead to global deterioration of quality of life due to disease-related symptoms which may in turn compromise the feasibility of second line treatment. To date, no clinical trial has demonstrated a significant improvement in quality of life with maintenance treatment as compared to observation. However, several studies have shown that global quality of life does not deteriorate, indicating the favorable toxicity profile of prolonged administration of single-agent cytotoxic or biological agents (28, 29).

Looking at maintenance with patient’s perspective suggests that majority will prefer maintenance over observation alone. In a pilot survey of 30 patients, Peeters et al. showed that metastatic NSCLC patients in general were in favor of maintenance and accepted either an OS benefit of at least several months, or better symptom control, in exchange for mild-to-moderate side effects. There was a slight preference for oral vs. intravenous administration (30).

## Metronomics as Maintenance Therapy

The concept of metronomic maintenance is not new. The success of this approach in ALL maintenance is the best and time-tested illustration (10). The effect of metronomic therapy on tumor microenvironment like inhibition of angiogenesis and immune modulation may be best utilized in maintenance setting when the disease burden is low. The “multi-targeted-metronomic-MTD chemo-switch (C-S)” model is the proof of concept for this strategy (31). It consists of administration of MTD chemotherapy followed by multi-targeted anti-angiogenic maintenance therapy. Recently, Vives et al. have also demonstrated that in pancreatic tumor models, C-S schedule of MTD and metronomic gemcitabine had the most favorable effect. They also showed that in contrast with standard doses of chemotherapy, metronomic and C-S gemcitabine both affect the pancreatic cancer stem cell (CSC) population (32). Similarly, a study of glioma subcutaneous xenograft model has shown the sensitivity of CSC population to metronomic cyclophosphamide administration (33). It has been proposed that CSCs can play a role in the resistance to conventional chemotherapy and radiotherapy, therefore targeting CSCs or the stem cell niche through antiangiogenesis with C-S metronomic approach opens a new avenue for clinical and preclinical research. In a recent review on maintenance therapy in NSCLC, Gerber et al. have suggested metronomic maintenance as a biologically rational approach (9).

## Clinical Experience of Metronomics as Maintenance

Although promising, metronomics as maintenance has not been extensively tested in clinical setting. Table 1 summarizes the clinical studies exploring role of metronomic therapies as maintenance. Most of these studies are small and single arm observations. However, a good safety profile and promising efficacy has been demonstrated by most of these studies. The results of a phase 3 trial (CAIRO-3) were presented at annual meeting of ASCO in 2013. After randomization of 558 patients and median follow-up of 33 months, improvement in PFS and OS was demonstrated with the use of maintenance metronomic dose of capecitabine along with bevacizumab (34).

**Table 1:** **(A) Published studies of metronomic maintenance; (B) ongoing clinical trials**.

Disease	Number of patients	Regimen used	Outcome	Toxicity	Reference

**(A)**					
NSCLLC	65 Maintenance 65 Observation	Paclitaxel 70 mg/m^2^ weekly 3 out of 4 weeks	PFS 38 vs. 29 weeks OS 75 vs. 60 weeks	One grade 3 or 4 toxicity – 45%	(35)
NSCLC	141 Received maintenance	Paclitaxel 70 mg/m^2^ weekly 3 out of 4 weeks	Median, TTP 33 weeks for arm 1 and 29 weeks for arm 2 (induction regimen arms)	Grade 4 neutropenia – 2.1%; Grade 3 toxicities: anemia (0.7%), neuropathy (2.1%), arthralgia (2.1%), fatigue (2.8%), dyspnea (2.1%), and abdominal pain (2.1%)	(36)
Various GI cancers	28 Patients	Capecitabine 1000 mg twice daily continuous	Efficacy not described	Only one grade 3 toxicity	(37)
Pediatric brain tumors (after HSCT)	10 Patients	Alternate cycles of 21 days of etoposide 50 mg/m^2^, cyclophosphamide 2.5 mg/kg/day, temozolomide 90 mg/m^2^/day, along with alt courses of celecoxib 100 mg BD, isotretinoin 100 mg/m^2^/day	8 out of 10 patients had stable disease at a mean duration of follow up of 20 months	Mild emesis (4/10), 1/10 had prolonged myelosuppression	(38)
Ovarian carcinoma	64 Patients	Paclitaxel 60 mg/m^2^ weekly for 21 weeks	3 year PFS 18%, 3 year OS 64%	Grade 2 neutropenia – 25.9%, Grade 2 sensorial neurotoxicity – 20.7%, Grade 2 motor neurotoxicity – 6.9%	(39)
Metastatic breast cancer	15	mC 1500 mg daily plus docetaxel 75 mg/m^2^ on day 1 of a 4-week cycle up to six cycles, followed by mC as maintenance	Objective response rate 41.7%, 1 (8.3%) CR, 4 (33.4%) PR, 6 (50%) SD clinical benefit of 66.7%, median time to progression 8.4 months	All toxicities occurred during D plus mC combination	(40)

**Disease and clinical setting**		**Regimen**	**Official title**	**Clinical trial identifier**	

**(B)**					
Breast cancer, ER, PR negative after adjuvant chemotherapy		CMM (cyclophosphamide 50/mg/day orally continuously for 1 year; methotrexate 2.5 mg/twice a day orally days 1 and 2 of every week for 1 year)	OT2-01-01: International Breast Cancer Study Group (IBCSG) trial 22-00	IBCSG 22-00	
Met CRC, after response to FOLFIRI + Bev		Capecitabine, celecoxib, and methotrexate	Metronomic chemotherapy with anti-angiogenic effect as maintenance treatment for metastatic colorectal carcinoma following response to FOLFIRI + bevacizumab: clinical and laboratory studies	NCT01668680	
Mets CRC after CAPOX–Bev		Capecitabine 625 mg/m^2^ bid daily continuously and bevacizumab 7.5 mg/kg iv q 3 weeks	Maintenance treatment with capecitabine and bevacizumab vs. observation after induction treatment with chemotherapy and bevacizumab in metastatic colorectal cancer (mCRC): the phase III CAIRO-3 study of the Dutch Colorectal Cancer Group (DCCG)	NCT00442637	
Met MBC		Capecitabine 500 mg/m^2^ three times daily on days 1–21 of each 3-week cycle and capecitabine 1000 mg/m^2^ twice daily on days 1–14 of each 3-week cycle	A randomized phase III study of metronomic vs. intermittent capecitabine maintenance therapy following first-line capecitabine and docetaxel therapy in HER2-negative metastatic breast cancer	NCT01917279	
Ovarian carcinoma, stage 3, after adjuvant chemotherapy		Cytophosphan tab 50 mg – 1 × 1 per day, continuous Celecoxib tab 200 mg – 1 × 2 per day, continuous Methotrexate tab 2.5 mg – 1 × 2 per day, 2 days weekly	Maintenance treatment for ovarian carcinoma in remission by an anti-angiogenic treatment strategy with metronomic/oral chemotherapy (cytophosphan combined with low-dose methotrexate) and COX-2 inhibition (celecoxib)	NCT01175772	

There are several ongoing clinical trials which are evaluating the role of metronomic maintenance in various combinations in colorectal cancer, breast cancer, and ovarian cancer (Table 1). The results of these trials will further clarify the utility of metronomic therapies as maintenance.

Three important aspects make metronomics appealing for maintenance: (1) favorable toxicity profile for long-term use. (2) The selection of low cost drugs for such regimens makes them widely practicable globally, especially in low and middle income countries (LMICs). (3) Their efficacy to target angiogenesis, CSCs, and immune modulation make them capable of overcoming resistance and delaying tumor progression.

## Potential Combination with Targeted Therapies

Combining MC with specific targeted therapies may eventually enhance the efficacy and specificity of a maintenance as demonstrated in several preclinical models and in the clinic (41–43). Noteworthy, the success of combination of targeted therapies with MTD chemotherapy has been variable and showed mostly failure when it was combined with anti-angiogenic TKIs in contrast to monoclonal antibodies (44–47). On the other hand, several preclinical and early clinical studies have shown that MC could be successfully combined with Pazopanib, an oral multi-kinase inhibitor showing encouraging activity in gynecological cancer (48) and pediatric solid tumors (41). MC, in that case, can be used to bridge the gap between biologicals and cytotoxics (48). The potential benefit of long-term use of targeted angiogenic inhibitors and MC can be gainfully utilized in maintenance.

Repositioning of several other drugs like celecoxib (49, 50), metformin (51, 52), propranolol (53, 54), valproic acid (55), atorvastatin (56), and itraconazole (57) which have shown anticancer properties in several preclinical, epidemiological, and clinical settings, can also be considered along with metronomic regimens for long-term use.

## Global Oncology Perspective

Ever-increasing cost of cancer treatment is a great public and political concern even in the developed world. In LMICs, where most of the new cancer cases and cancer deaths occur, access to most of modern treatment modalities is beyond imagination. High income countries’ standards of care, although appealing, are out of reach for LMICs because of their cost, toxicities, and the complex infrastructure and technology needed. As we have proposed earlier, metronomics may be a promising alternative strategy for the improvement of cancer care in LMICs (58). This treatment strategy seems even more promising in maintenance setting where long-term administration is required and can be more acceptable because of favorable toxicity profile and affordable cost.

## Conclusion

The field of metronomics still is *terra incognita* even after more than a decade since conception. The selection of best metronomic regimen for individual disease, the ideal combination and scheduling, predictive bio-markers, and the timing of institution are certain areas which still remain unanswered. Apart from relapse/refractory settings where this therapy has been tested mostly and showed meaningful activity, there is enough scientific rationale for their evaluation as maintenance strategy. Preclinical work for selection of drugs to be used, identification of predictive bio-markers, and rationale combination with other biological agents are some areas which warrant further research.

## Conflict of Interest Statement

The authors declare that the research was conducted in the absence of any commercial or financial relationships that could be construed as a potential conflict of interest.
